# Current and Future Molecular Diagnostics of Tick-Borne Diseases in Cattle

**DOI:** 10.3390/vetsci9050241

**Published:** 2022-05-21

**Authors:** Kathryn Garcia, Mina Weakley, Tram Do, Sheema Mir

**Affiliations:** College of Veterinary Medicine, Western University of Health Sciences, Pomona, CA 91766, USA; kathryn.garcia@westernu.edu (K.G.); mina.weakley@westernu.edu (M.W.); tram.do@westernu.edu (T.D.)

**Keywords:** ticks, bacterial, viral and protozoal tick-borne diseases, multiplex molecular diagnostics, PCR, cattle

## Abstract

Ticks and tick-borne diseases such as babesiosis, anaplasmosis, ehrlichiosis, Lyme disease, Crimean Congo hemorrhagic fever, and Rocky Mountain spotted fever pose a significant threat to animal and human health. Tick-borne diseases cause billions of dollars of losses to livestock farmers annually. These losses are partially attributed to the lack of sensitive, robust, cost effective and efficient diagnostic approaches that could detect the infectious pathogen at the early stages of illness. The modern nucleic acid-based multiplex diagnostic approaches have been developed in human medicine but are still absent in veterinary medicine. These powerful assays can screen 384 patient samples at one time, simultaneously detect numerous infectious pathogens in each test sample and provide the diagnostic answer in a few hours. Development, commercialization, and wide use of such high throughput multiplex molecular assays in the cattle tick-borne disease surveillance will help in early detection and control of infectious pathogens in the animal reservoir before community spread and spillover to humans. Such approaches in veterinary medicine will save animal life, prevent billions of dollars of economic loss to cattle herders and reduce unwanted stress to both human and animal health care systems. This literature review provides recent updates on molecular diagnostics of tick-borne pathogens and discusses the importance of modern nucleic acid high throughput multiplex diagnostic approaches in the prevention of tick-borne infection to livestock.

## 1. Introduction

Ticks are obligate blood feeding arthropods. They carry bacteria, helminths, protozoa, and viruses that are pathogenic to their vertebrate hosts, including humans, domestic and wild animals. Ticks transfer pathogens from their gut to the host bloodstream by their saliva [1]. Different categories of tick-borne pathogens cause diseases in either human or domestic animals or both. On a global scale, the economic loss caused by tick-borne diseases is staggering. Every year, ticks and tick-borne pathogens cause around USD 13.9–19.7 billion in losses in the United States (USA), which includes approximately three billion in losses in cattle tick infestations alone [1,2]. Cattle and livestock farming is important for the economic and sociocultural wellbeing of any country. Livestock supports the food supply, family nutrition, family income, asset savings, soil productivity, livelihoods, transport, agricultural traction, agricultural diversification and sustainable agricultural production, family and community employment and income, ritual purposes, and social status [3,4]. National cattle herders have expanded in countries with the highest cattle inventories such as Brazil, Australia, the USA, India, Argentina, and the European Union. On average, the USA imports one million cattle annually from Mexico, which are often affected by ticks and tick-borne diseases [5]. Ticks transmit many diseases [6] to domestic and livestock animals which includes viral diseases such as Crimean-Congo hemorrhagic fever and tick-borne encephalitis virus, bacterial diseases such as Q fever, borreliosis and relapsing fever, protozoal diseases such as theileriosis and babesiosis, and rickettsial diseases such as anaplasmosis and ehrlichiosis.

Tick-borne pathogens circulate in enzootic cycles, alternating between ticks and suitable animal hosts. Tick-borne diseases of livestock increase workloads by necessitating the adoption of preventive measures in controlling the disease. In addition, the financial stress to livestock owners due to animal loss contributes to the psychosocial stress, affecting their quality of life. Better global control of tick-borne diseases of livestock and their vectors would contribute substantially to improved meat and milk production [7]. There are over 60 tick-borne agents that may be pathogenic to livestock, however few are recognized as being of economic significance [8]. In addition, it is now well understood that ticks may be co-infected with more than one pathogen and transmit multiple pathogens simultaneously while taking a blood meal from their hosts [9,10].

The overall impact of tick-borne diseases on livestock operations is likely higher because it is difficult to measure the impact of parasites on cattle weight, reduced milk production, aborted calves or other health problems that reduce production. Decades after the identification of causative agents for tick-borne diseases, we still have limited tools to manage the impact of losses incurred by tick-borne diseases. Although the use of antibiotics against bacterial agents has helped reduce certain diseases, animals remain susceptible to reinfection [11]. Likewise, the use of dipping techniques for cattle to kill the ticks has helped, but the emergence of acaricide resistance has become a problem [12]. Although live attenuated and recombinant vaccines are considered as preferred measures in tick-borne disease control [13], none of them have resulted in sterile immunity. Furthermore, the difficulty in isolating and identifying various infectious pathogens have made it a complex problem. A few more drivers of change in these disease dynamics include climate and ecosystem changes, globalization, with movements of people and animals, and an increasing demand for livestock products [14], which is likely to have increasing public health implications [15]. The costly serology based (singleplex) diagnostic approaches discourage the cattle herders from screening their cattle for potential tick-borne infections [16]. Moreover, the homology of epitopes between different infectious pathogens or their strains can generate cross-reactive antibodies that can provide false positive test results [17].

In the USA, the APHIS’ (Animal and Plant Health Inspection Service) cattle health surveillance system protects cattle from incursions of foreign, emerging, and certain endemic diseases. The rapid identification and prompt implementation of countermeasures can save billions of dollars for the domestic and international trade market. The APHIS cattle surveillance system helps to minimize production losses and maintains market viability [14]. This review briefly summarizes the clinical manifestations, transmission, surveillance, and diagnostics of tick-borne animal diseases caused by bacterial, viral, and parasitic infections to the cattle and domestic animals in the United States and abroad. In addition, this review article discusses the significance of multiplex diagnostic approaches for the simultaneous detection of multiple tick-borne agents in a single test sample in a short turnaround time. Such modern diagnostic tools will revolutionize the identification of tick-borne illnesses in cattle by providing an accurate diagnostic answer in a timely fashion, minimizing economic losses to cattle owners. Currently, there are no standardized multiplex molecular diagnostics assays that are commercially available for tick-borne diseases. Nucleic acid multiplex molecular diagnostic tools will thus be of paramount importance in controlling the disease spread and can be used as an effective tool for surveillance studies.

## 2. Protozoal Agents

### 2.1. Babesiosis

Babesiosis poses major health and management problems to cattle and small ruminants. There is a growing interest in this protozoan parasite because of its involvement in human infection. *Babesia* and its close members together with *Theileria* are called Piroplasms because of the pear-shaped appearance of the parasites in infected erythrocytes (piroplasmosis). There are more than 100 species of tick transmitted protozoal pathogens of the genus *Babesia* that infect a variety of vertebrate hosts. The parasite attacks and damages host erythrocytes much like malarian protozoa (*Plasmodium)*. *Babesia* species (spp.) may be transmitted by Ixodid ticks of genera *Rhipicephalus,* which can be either transovarial (from one generation to the next via the egg) or transstadial (from egg to larva) [18]. For cattle, two organisms, *Babesia bovis* and *Babesia bigemina* collectively cause “Cattle tick fever”. These organisms have a worldwide distribution in tropical and subtropical regions, and in the USA, they have caused huge economic losses to the cattle industry in the late 1800s and 1900s [18]. Bovine babesiosis is often observed in adult cattle with clinical manifestations such as fever, anorexia, anemia, along with several neurological symptoms. Mild cases can recover without treatment, although survivors may remain weak for a long time. Severe cases of bovine babesiosis are treated with antiparasitic drugs. In addition, vaccines are also commercially available for bovine babesiosis [2].

PCR-based diagnostic approaches are gaining acceptance [19], and recently the rapid detection of *Babesia* spp. specific DNA sequences by nested PCR (Table 1) have been used for epidemiological studies worldwide, targeting *B. bovis* rhoptry-associated protein-1 (BbovRAP-1), *B. bovis* spherical body protein 2 (BboSBP2), *B. bovis* spherical body protein 4 (BboSBP4), *B. bigemina* Apical Membrane Antigen-1 (BbiAMA-1) and *B. bigemina* rhoptry-associated protein-1a (BbiRAP-1a) [20]. Since this article is focused on PCR based molecular assays, we have incorporated the non-molecular assays with appropriate references in Table 1 for the readers who intend to make a comparison between the PCR based molecular assays and the non-molecular assays available for these pathogens.

### 2.2. Theileriosis and East Coast Fever

About 15 or more parasitic protozoa species of the genus *Theileria* are transmitted by ixodid ticks to domesticated ruminants that cause the most significant economic losses [33]. In the mammalian host, parasites infect leukocytes and erythrocytes. In the arthropod vector they develop in gut epithelial cells and salivary glands. Some *Theileria* species tend to circulate with few or no clinical signs, but others can cause serious illnesses with high morbidity and mortality rates [34]. The two organisms with the greatest economic impact in cattle are *Theileria parva* and *Theileria annulata*, which cause East Coast fever/corridor disease and tropical theileriosis, respectively. East Coast fever/corridor disease occurs in sub-Saharan Africa and is characterized by fever, generalized lymphadenopathy, anorexia, loss of condition along with nasal discharge and or diarrhea observed in some animals. Tropical theileriosis (*T. annulata*) generally resembles East Coast fever, but these parasites also destroy red blood cells, causing anemia and, in some cases, jaundice or hemoglobinuria. Another species, *Theileria orientalis,* is widespread and has been reported from Europe, Asia, Africa, North and South America, Australia, and New Zealand [33,35,36]. *Theileria sinensis* has been documented in Asia (China) and Africa [37]. *Theileria* transmission via ticks occurs transstadially, and transovarial transmission is not thought to occur [38]. Disinfection is not important in the control of theileriosis. Treatment with antiparasitic drugs is helpful in early stages of infection. The mortality rate for tropical theileriosis is reported to be 40–90% in newly introduced cattle but less than 5% in some indigenous animals [33].

Diagnostic testing involves finding piroplasms or schizonts using PCR, which has had a profound impact on the identification of tick-borne pathogens. PCR can identify *Theileria* in the blood of both carriers and clinical cases (Table 1) [39]. Molecular diagnostics, however, are not widely available in comparison to serological tests even though they are more sensitive and could provide crucial insight in studying the role of carrier animals as a source of infection [40,41]. Recently in the USA, the detection of the *T. orientalis* genotype Ikeda was found to be a risk for the cattle industry in Virginia. The Animal Health Diagnostic Center (AHDC) at Cornell University is now offering a PCR test for *T. orientalis* [42]. The most pathogenic species in cattle and small ruminants, such as *T. annulata, T. parva, Theileria lestoquardi, Theileria luwenshuni* and *Theileria* *uilenbergi* are all exotic to the USA and must be reported to state or federal authorities immediately upon their diagnosis or suspicion [43], and outbreaks have often occurred after animals were moved from one region to another.

## 3. Bacterial Agents

### 3.1. Anaplasmosis

Anaplasmosis is a tick-borne disease of cattle caused by the parasite *A. marginale* that leads to the destruction of red blood cells. Anaplasmosis is spread by the mechanical transfer of blood from an infected animal to a susceptible one or by using contaminated instruments. Researchers have demonstrated that the Pacific Coast tick and the Rocky Mountain wood tick (*Dermacentor* spp.) can also be transmitters of this disease [21]. Anaplasmosis in cattle has a worldwide distribution, but the disease is most common in tropical and subtropical areas. Infected animals become anemic, weak, lethargic, go off feed and develop fever. The mucous membranes become pale and possibly yellow from the waste products of red blood cell destruction. A characteristic symptom of anaplasmosis is that the urine will not be red or brown as with leptospirosis [21]. Cattle of all ages are susceptible to infection, and the severity of disease increases with age. Furthermore, infected animals become persistent carriers for life [21,44]. Chemicals such as acaricides are widely used to manage tick infestations in addition to the use of vaccinations [6]. Yet, intensive use of such chemicals has resulted in the emergence of drug resistant tick populations [45]. Cattle tick fever (CTF) is a complex of disease caused by the Rickettsia *A. marginale* (anaplasmosis) and the protozoa *B. bovis* and *B. bigemina* (babesiosis) and is transmitted by *Rhipicephalus* (*Boophilus*), causing significant economic losses worldwide [44,46,47].

PCR has been used recently for diagnostics in pre-symptomatic or carrier animals (Table 1) [21]. In many endemic areas, nested PCR’s have been used for the diagnosis of carrier cases of anaplasmosis [48,49]. A multiplex PCR followed by the magnetic capture method using species-specific oligonucleotides to detect six Anaplasma/Ehrlichhia species has been recently used in cattle [50].

In the USA, the lack of anaplasmosis seroprevalence surveys makes it difficult to estimate the accurate production losses incurred to the cattle industry [51]. In addition, the lack of research about the susceptibility of wild ruminants with tick-borne diseases along with the lack of validated molecular diagnostic tests for many of these pathogens have led to a gap in the scientific literature. Better, sensitive, and improved nucleic acid diagnostic assays will positively contribute towards the early detection of these elusive pathogens that are responsible for the most devastating tick-borne diseases of the cattle [49].

### 3.2. Lyme Borreliosis

Lyme borreliosis is the most common vector-borne infection in humans in the USA. According to the Center of Disease Control and Prevention (CDC), Lyme borreliosis occurs in domestic animals and cattle. *Borrelia burgdorferi*, a spirochete transmitted by *Ixodes* ticks, is the causative agent [24]. These ticks are geographically distributed in temperate regions of the northern hemispheres. In cattle, borreliosis is usually a herd problem [24]. Clinical signs of borreliosis may include fever, stiffness, swollen joints, and decreased milk production. Disease outcomes of borreliosis are chronic weight loss, laminitis, and abortion. Lyme borreliosis is diagnosed based on the history of exposure, clinical signs, identification of spirochetes in the test sample, and the exclusion of other diseases [52]. PCR tests are available which may be used to detect the genomic DNA in the tissue, cerebrospinal fluid (CSF) or ocular fluid, which involves more invasive sampling. PCR tests have shown *B. burgdorferi* (Table 1) to be genetically diverse, and it is represented by more than 20 different species. Realtime PCR detected *Borrelia* strains in samples of the synovial fluid and milk of two cows from two different herds in Switzerland [53]. In the USA, many veterinary diagnostics labs like North Dakota State University Veterinary Diagnostic Laboratory, which is a part of the National Animal Health Laboratory Network (NLHLN), offers PCR testing using the flagellin gene of *B. burgdorferi* [54]. New research studies have focused on the elevated immune response to tick-borne pathogens and will be helpful in the development of new vaccines [55].

### 3.3. Ehrlichiosis (Heartwater)

Ehrlichiosis, also known as Heartwater or Cowdriosis, is caused by *Ehrlichia ruminantium* (*Cowdria ruminantium, Rickettsia ruminantium*). Cattle are affected worldwide, primarily in Africa and the Caribbean. It is considered an agricultural biothreat with no vaccine available at present. Ehrlichia infection in cattle is often unreported because it may be associated with subclinical infection (carrier state) [56]. Clinical pathological changes in ehrlichiosis are highly variable and infected cattle develop fever, become disoriented and show signs of motor disorders like abnormal walking and muscle twitching, anemia and lymphadenitis. There is high mortality with animals, and death occurs within 36 to 48 h in the acute form of the disease. The ticks *Amblyomma variegatum* and *Rhipicephalus appendiculatus* are well characterized vectors for ehrlichiosis [57]. Heartwater presents serious economic and social problems in endemic areas of Africa. Methods used for the control of heartwater include tick control, farming with resistant animal stock, antibiotic treatment, and immunization [57]. In ruminants, PCR based molecular tests are available. The PCR based molecular test specifically detects the pCS20 gene in the test sample (Table 1). One major problem in the development of nucleic acid-based assays in the pCS20 regions of different *E. ruminantium* isolates are the presence of sequence polymorphisms, mostly single nucleotide polymorphisms. Sequence homology with *Ehrlichia chaffeensis* and *Ehrlichia canis,* on the other hand, causes cross reactivity, resulting in false positives with the same assay. Therefore, the need to design new molecular assays for better specificity and inclusivity of *E. ruminantium* populations is necessary [26].

## 4. Viral Agents

### 4.1. Tick Borne Encephalitis

Tick-borne encephalitis (TBE) is a viral infectious disease (*Flavivirus* genus, family *Flaviviridae*) that occurs in many parts of Europe and Asia [58]. In humans, it involves the central nervous system and often results in long term neurological symptoms including severe encephalitis and myelitis. The virus is transmitted by the bite of infected ticks (*Ixodes ricinus* and *Ixodes persulcatus*) found in woodland habitats [59]. In domestic species such as dogs and horses, TBE infection may manifest with clinical signs similar to those seen in severe human cases, otherwise in ruminants such infections are typically asymptomatic [60,61]. However, such asymptomatic infection poses a greater risk to humans. For example, the presence of Tick-borne encephalitis virus (TBEV) in the milk of infected ruminants can serve as a source of human TBE infection [62]. Over the past decade, the number of annual TBE cases has increased in TBE endemic regions as well as in new emerging areas [63]. TBEV infection occurs via the consumption of unpasteurized dairy products [64]. TBEV can survive in milk for up to two weeks at 4 °C with only a modest decrease in titer [65]. Standard preventative measures, therefore, include pasteurization of milk and dairy to prevent the risk of alimentary TBEV infection in humans. Recent advances in prevention also includes a vaccination drive of selected domestic animals to avoid the development of severe TBE symptoms or to decrease the risk of alimentary infection in humans [66,67].

Since the infection of ruminants is relevant for public health, the early diagnosis of TBEV becomes important. The serologic diagnosis of TBEV lacks specificity and requires additional verification by a serum neutralization test (SNT) [31,32] (Table 1). Nucleic acid tests that detect viral RNA in target specimens are available (Table 1). Aside from singleplex PCR, multiplex assays may be used as screening tests. Such broad-panel systems for the simultaneous detection of nine tick-borne pathogens in humans are currently available for research use only [68]. The big gaps in knowledge about immunity in animals remains a consistent problem [69].

### 4.2. Crimean-Congo Hemorrhagic Fever

Crimean Congo hemorrhagic fever is a concerning global viral illness in both animals and humans. The disease has a characteristic sudden onset in humans. It is caused by the Crimean Congo hemorrhagic fever virus (CCHFV), a tick-borne *Nairovirus* in the order *Bunyavirales* [70,71]. Ixodid ticks, especially from *Hyalomma* genus, are both the reservoirs and vectors for CCHFV [72]. Wild and domestic animals such as cattle, sheep and goats serve as amplifying hosts for this virus, especially in the warm summer months when tick populations are on the rise [73]. Infected livestock have mild or no symptoms and often become the starting point of outbreaks in people. Clinical signs have been demonstrated in experimental settings like transient fever in calves [74]. Although CCHFV is not pathogenic to ruminants, its infection can trigger unwanted animal slaughter. Transmission to humans occurs through contact with infected ticks or animal blood and tissues immediately and after slaughter [75]. The human-to-human transmission of CCHFV is well known [76]. CCHFV infection causes global health problems due to the lack of Food and Drug Administration (FDA) approved vaccine and effective therapeutic agents for both humans and animals [77]. Both the CDC and World health Organization (WHO) reported a 40% fatality rate in CCHFV infected humans [78]. Therapeutic intervention of the CCHFV life cycle in either tick vectors or infected animal and human hosts is a novel approach to control the morbidity of this viral illness. It is difficult to control the CCHFV infection of animals and ticks because the tick-animal-tick cycle often goes unnoticed, and the infection in domestic animals is usually not that evident. Since the tick vector is so widespread, the tick control using acaricides seems the most viable option for well-managed livestock facilities.

The laboratory diagnosis involves the detection of viral genome in the patient sample by Reverse Transcription-PCR (RT-PCR) and detection of antibodies against the CCHFV in patient serum by ELISA [29]. Since the antibody production can be delayed, the detection of viral genome by RT-PCR remains the preferred diagnostic approach for CCHFV infection in animals (Table 1) [29].

### 4.3. Severe Fever Thromocytopenia Syndrome

Sever fever with thromocytopenia syndrome (SFTS) is caused by a RNA virus-SFTS virus (SFTSV), a new member of the genus Phlebovirus [79]. The disease is an emerging zoonotic disease caused by ticks (*Rhipicephalus* and *Ixodes* spp.) and is characterised by fever, thrombocytopenia and leukopenia [79]. Since the first report from China, the disease has been on the increase and has been found in other parts of East Asia (Japan and South Korea) with a high mortality rate and no treatment [80,81]. Domestic animals have been shown to be major reservoirs in transmission of the disease [82]. Although ELISA [83] and IFA’s [84] are also frequently used for diagnostic testing, the nucleic acid molecular assays remain the preferred diagnostic approach. Molecular diagnostic tools using a highly sensitive and quantitative real-time PCR assay for the detection of S, L and M segments of the viral genome in the test sample remains the reliable diagnostic method for SFTSV infection [85]. SFTV RNA has been detected in blood samples of several animals, with the highest carriage rate in cattle (26.31%), with no evidence that the virus can cause disease in animals [86,87].

## 5. Multiplex Diagnostics of Tick-Borne Pathogens in Cattle

The quick identification of infectious pathogens in infected animals is profoundly important, as the pathogen can spread in the population and create a global health concern of pandemic origin for both humans and animals. One infected animal can spread the tick-borne infection to the entire herd, especially in the summer months when tick populations are on the rise, causing significant loss to herd owners and the loss of animal life [1,2]. The situation can worsen if the infectious pathogen spreads to humans, which can trigger the unwanted slaughter of the infected herd to bring the infectious disease under control. The singleplex diagnostic strategies such as culturing of infectious bacterial, viral, and fungal pathogens or identification of pathogen specific antigens or antibodies in the patient serum samples lack sensitivity and are time consuming. Often, the antibody response to the infectious pathogen is dysregulated and the identification of a pathogen-specific antigen or antibody becomes complicated during the early stages of illness [88,89,90]. The frequently used serologic assays for the diagnosis of tick-borne disease include ELISA, IFA, and western blot [91,92]. However, their diagnostic accuracy is affected by numerous limitations. For example, the recommended two-tiered diagnostic approach for Lyme disease consist of ELISA followed by western blot. This can detect <40% of patients with early disease and can result in up to 28% of IgM western blots yielding false positive results [93]. Similarly, the accuracy of IFA, which is recommended for the detection of *Babesia, Anaplasma, Ehrlichia* and *Rickettsia,* can vary widely among diagnostic laboratories primarily due to the lack of standardized antigenic targets, cross reactivity, and the subjective establishment of positivity thresholds [94]. These limitations, especially the huge cost and poor sensitivity of old singleplex serological diagnostic approaches, can discourage the herd owners from screening their animals for tick-borne infectious diseases. In addition, coinfection with more than one tick-borne pathogen has been reported in animals [9,10]. The coinfections will require multiple singleplex screening tests on the same sample. Moreover, the screening of numerous tick-borne pathogens in huge reservoir populations for surveillance studies is extremely difficult and costly by singleplex diagnostics approaches.

The first multiplex array-based serologic assay called Tick-Borne Disease Serochip (TBD-Serochip) was developed by employing an extensive range of linear peptides that identify key specific immunodominant epitopes [95]. The assay discriminates the antibody responses to eight major tick-borne pathogens found in the United States, including *A. phagocytophilum*, *B. microti, B. burgdorferi, B. miyamotoi, E. chaffeensis, Rickettsia rickettsii,* Heartland virus and Powassan virus [95]. The major limitation of this interesting platform is that it displays only linear peptides and may miss the conformation determinants or non-protein epitopes important for pathogenesis. Although this novel serological platform holds promise for further development as a diagnostic tool, it will need further development and validation by comparing their output test results with existing serologic assays that are primarily used for the diagnosis of tick-borne diseases. In addition, the cost may become a limiting factor for the widespread use of such peptide array-based multiplex diagnostic assays in the veterinary field. The development of small multiplex assays, detecting antibodies against the three surface proteins (OspA, OspC and OspF) of *B. burgdorferi* in canine [96] and horse [97], have also been developed. Similar multiplex assay detecting antibodies against five surface proteins of Lyme disease have been reported, where five *B. burgdorferi* antigens were combined into a fluorescent cytometric bead-based assay for detection of specific IgG antibodies [98]. However, the lower sensitivity and cross reactivity of antibodies have created hurdles in the rapid development of serology-based multiplex diagnostic approaches.

In comparison, the sensitive and cost-effective PCR-based diagnostic approaches can detect even a single pathogen in the test sample with high specificity, and they are thus preferred in the diagnostic industry for their reliable test results, especially for the determination of active infection. With this ever-changing world around us, we need to develop reliable diagnostic approaches that can detect multiple pathogens with higher specificity and sensitivity from the same test sample in veterinary medicine. In addition, reporting and sharing of the diagnostic test results with the concerned authorities should be made simple and easy. Surprisingly, the efficient and cost effective multiplex nucleic acid-based diagnostic tests have not been significantly developed in veterinary medicine [99] but have taken bigger leaps in human medicine [100,101]. For example, modern multiplex diagnostic technology has helped clinicians to accurately detect the target pathogen in the infected human patients that helped in the initiation of accurate treatment plans in a timely fashion, resulting in a favorable disease outcome [102,103]. There is a greater need for the development and commercialization of these tests, since they can provide greater information beyond species identification such as drug resistance, strain divergence, virulence, and the origin of isolates. Unlike serology-based assays, the PCR- based nucleic acid diagnostic assays provide clear insight about the active infection in the patient. Nucleic acid based multiplex diagnostic assays are highly sensitive, cost effective, and have a very short turnaround time. These assays can screen 96 to 384 samples of a cattle herd for almost all-important tick-borne pathogens and provide the diagnostic answer in just 4 to 5 h. These powerful assays can be very helpful in the surveillance studies for the identification of tick-borne pathogens in different tick and animal populations in various geographical areas of the world to gauge prior insights about the potential exposure to a tick-borne infection. Their routine use in the screening of any infectious disease in cattle will revolutionize cattle farming by minimizing the loss to cattle owners.

## 6. Advantages of the Nucleic Acid Multiplex Diagnostic Approaches

The advantages of the nucleic acid multiplexing platform are (i) the reaction kinetics of multiplex platforms such as the platforms used by barcoded magnetic bead technology (BMB) are rapid, which is favored by the mixing of barcoded magnetic beads with the test samples in liquid suspension [100,101]. In comparison, the microarray chip-based formats are more expensive and have slower reaction kinetics; (ii) The signal to background ratio of at least 10,000 for BMB technology is markedly high [100,101], which makes the sample detection easy, clear and highly reliable; (iii) The turnaround time of 4 to 5 h from sample collection to the final delivery of test results for most nucleic acid multiplex assays demonstrates the overall efficiency of this diagnostic approach. This is extremely important in controlling the spread of an infectious disease, which requires the rapid identification and isolation of the positive cases; (iv) The nucleic acid multiplex diagnostic assays are highly sensitive, as they can detect even a single copy of the genome of the infectious pathogen in the test reaction. This helps in the diagnosis at the early stages of illness, which ultimately helps in preventing the spread of the infectious disease; (v) The cost-effective nature of nucleic acid multiplex diagnostic approaches makes them affordable for most patients. Such assays can help the herd owners to screen the entire heard for numerous infectious pathogens at a minimum cost; (vi) The high throughput processing of 96 to 384 samples at one time is of critical significance during a pandemic when an overwhelming number of patient samples burden the diagnostic centers; (vii) Simultaneous detection of all medically important tick-borne pathogens such as *A. phagocytophilum, B. microti*, *B. burgdorferi*, *B. miyamotoi*, *E. chaffeensis*, *Rickettsia rickettsii*, TBEV, ASFV and CCHFV, in a single patient sample, will provide the conclusive diagnosis in the shortest time span and will help the veterinarian to initiate the therapeutic or preventive measures in timely fashion; (viii) The multiplex diagnostic approaches identify co-infections which can otherwise remain undetected by classical old diagnostic approaches; (ix) The ability to detect direct pathogens in an extending diagnostic window for many days as compared to serology [68]; and (x) The high throughput multiplex diagnostic approach is a personalized diagnostics approach that will guide the implementation of public health measures by quick identification as well as the quarantining of infected patients along with monitoring community exposure rates. The potential for multiplex diagnostics of emerging pathogens is huge, and it is becoming a preferred method of diagnosis among clinicians, especially in human medicine [102,104].

In the past decade, multiplex molecular testing in the veterinary field is catching up to those methods routinely practiced in human medicine and is facilitating the ability to perform surveys determining the prevalence of common tick-borne pathogens in cattle. We scouted the literature to report these newly developed molecular assays here. The latest is the development and validation of a novel six-plex assay detection by magnetic capture method using species-specific oligonucleotides to detect six *Anaplasma/Ehrlichia* spp. pathogens in canine, bovine, caprine and ovine blood samples. The assay uses a16S rRNA gene-based real time quantitative PCR assay combined with Luminex xMAP hybridization technology [50]. Another PCR-based multiplex assay using heat shock protein (*gro*EL), the citrate synthase gene (*glt*A) and the 18s rDNA gene for *Anaplasma*, *Babesia* and *Theileria* spp. was developed in Malawi to study the burden of tick-borne pathogens [105]. Another multiplex assay for diagnosis of the co-detection of tropical theileriosis, bovine babesiosis and anaplasmosis was developed using conserved sequences of cytochrome b gene, erythrocyte surface antigen and major surface protein [106]. In one more study, clinically healthy cattle with no signs of apathy, jaundice, anemia, or hemoglobinuria were tested using a multiplex PCR test that detects major surface proteins of *A. marginale, B. bovis* and *B. bigemina*. About 53.5% of the study population was found to carry one or more pathogenic agents [107].

Although multiplex PCR has many advantages, its disadvantages, however, cannot be ignored. Multiplex PCRs are complex, and they require rigorous testing, optimization and the troubleshooting of various PCR components, which often can be very difficult. The optimization of primers can pose several difficulties at times due to the preferential amplification of several targets or nonspecific target amplification [108]. A stepwise matrix style approach for primer mixing and testing is often used where a few primers for two or three pathogens are tested and the combination that shows the best sensitivity is then chosen in multiplex. Primers that detect multiple strains are usually selected in order to ensure the identification of as many strains of the target species as possible. However, since there are many primers and amplicons, the possibility of getting cross-hybridized with unintended targets increases almost exponentially. Alterations of PCR components like buffers and polymerases is also done using a trial-and-error approach during feasibility and development testing of a multiplex assay. External and internal quality controls like negative specimens and confirmed positive controls must be used to develop a robust assay [104]. All of the above limitations are addressed during the development and validation of multiplex assays, making the process tedious. However, the availability of multiplex assays in underdeveloped countries continues and remains a disadvantage, with many multiplex assays requiring special instruments and trained personnel [109].

## 7. Conclusions

Tick-borne diseases afflict cattle from temperate to tropical regions of the world and the economic losses due to them are considerable, especially in high-yielding dairy breeds and beef cattle due to reduced productivity [110]. Ticks transmit a greater diversity of viral, bacterial, and protozoan diseases than any other arthropod vector on earth [111]. More than 27 ecologically and epidemiologically distinct tick-borne diseases have been previously identified in the Western Hemisphere [112]. The CDC has reported 50,865 human cases of tick-borne diseases (tick-borne diseases surveillance data) in 2019, and this number has been gradually increasing for years [113]. Although 19 bacterial, protozoan, and viral agents have been implicated in tick-borne disease, *Borrelia burgdorferi*, the causative agent of Lyme disease, alone accounts for an estimated 300,000 annual cases of tick-borne disease in both humans and animals [114]. The number of annual tick-borne infections is expected to increase in future due to climate change as the warmer weather favors tick growth. Every year, ticks and tick-borne infections cause around USD 13.9–19.7 billion in losses in the United States, and this is expected to increase in future due to global warming [2,115]. The tick-borne infection of cattle origin spilling over to human populations can trigger unwanted animal slaughter, causing huge economic losses to cattle owners. The widespread nature of tick-borne infections in diverse geographical areas and their accompanying huge economic losses are mostly due to lack of efficient, sensitive, and cost-effective diagnostic approaches in surveillance studies. The frequently used protein based singleplex serologic assays for the diagnosis of tick-borne disease including ELISA, IFA, and western blot have many intrinsic limitations such as poor sensitivity, they are uneconomical, incompetent, and they have long turnaround times. These limitations discourage herd owners from using these assays for routine testing and surveillance studies to gauge prior insights about the potential exposure to tick-borne diseases. As a result, the infection goes unnoticed and infected animals act as silent reservoirs for the pathogen and can spread the disease among the population, ultimately magnifying the economic loss, and increasing the chances of potential spillover to human populations. The need of the hour is to develop highly sensitive, cost effective and efficient nucleic acid based multiplex diagnostic assays that can detect all important tick-borne pathogens in a single test sample and provide the diagnostic answer in 4–5 h. Such assays have been developed in human medicine but are still missing in animal medicine, although no technological or methodical limitations for the development of such assays exist in veterinary science. Such multiplex assays can be effectively used for both surveillance studies and routine testing of the cattle herd. The extremely sensitive nature of these PCR-based multiplex assays will be helpful in the detection of the pathogen at the early stages of illness, enabling the initiation of mitigation strategies and the prevention of community spread. The high throughput and short turnaround time of the nucleic acid multiplex assays will encourage the cattle herders to perform the surveillance studies to gauge prior insight about the potential exposure to tick-borne agents in any geographical area. The future research should focus on the development of countermeasures that will be helpful in controlling the tick-borne disease within the animal or tick reservoir before community spread or spillover to human population occurs. Such countermeasures include surveillance using multiple diagnostic molecular tests which will be extremely beneficial to both human and animal health and will dramatically reduce the economic losses incurred by tick-borne infections. Global warming will enforce the rapid development of both nucleic acid multiplex diagnostic assays and countermeasures in the near future to prevent the overwhelming spread of tick-borne infections to both cattle and human populations.

## Figures and Tables

**Table 1:** Available diagnostic tests for tick-borne pathogens in cattle.

Disease	Pathogen Name	Available Diagnostic Test	References
Anaplasmosis	*Anaplasma phagocytophilum*	Microscopy, Serology, PCR ^#^	[21]
	*Anaplasma marginale*		
Babesiosis	*Babesia bovis, Babesia bigemina*	ELISA *, IFA **, PCR	[19,20]
Borreliosis &	*Borrelia miyamotoi, Borrelia theileri*	Serology,	
Lyme disease	*Borrelia burgdorferi*	PCR, IFA, ELISA	[22,23,24,25]
	*Ehrichia ruminantium*		
Ehrlichiosis	*Ehrlichia bovis*	Serology, ELISA, PCR	[26]
Theileriosis &	*Theileria orientalis, Theileria parva*	PCR, Serology, ELISA, IFA	[27,28]
East coast fever	*Theileria annulata*		
CCHF ^=^	CCHF virus	PCR, ELISA	[29]
TBE *^†^*	Tick-borne encephalitis virus	ELISA, SNT ^‡^, IHC ^⊥^	[30,31,32]

* (ELISA-enzyme—linked immunosorbent assay); ^#^ (PCR—Polymerase chain reaction); ** (IFA—Immunofluorescence assay); ^†^ (TBE—Tick-borne encephalitis); ^‡^ (SNT—serum neutralization test); ^=^ (CCHF—Crimean Congo Hemorrhagic Fever); ^⊥^ (IHC—Immunohistochemistry); Note: The non-molecular assays shown in this table are for the readers who intend to compare the PCR based molecular assays with the non-molecular diagnostic approaches using the citations provided above.

## Data Availability

Not applicable.
